# Dietary Impact on Postprandial Lipemia

**DOI:** 10.3389/fendo.2020.00337

**Published:** 2020-07-07

**Authors:** Lutgarda Bozzetto, Giuseppe Della Pepa, Claudia Vetrani, Angela Albarosa Rivellese

**Affiliations:** Department of Clinical Medicine and Surgery, University of Naples “Federico II”, Naples, Italy

**Keywords:** postprandial lipemia, triglyceride-rich lipoprotein, triglyceride concentrations, diet, dietary components, dietary patterns

## Abstract

Abnormalities in postprandial lipemia (PPL), particularly those related to triglyceride-rich lipoproteins, are considered an independent cardiovascular risk factor. As diet is known to be one of the main modulators of PPL, the aim of this review was to summarize and discuss current knowledge on the impact of diet and its components on PPL in humans; specifically, the impact of weight loss, different nutrients (quantity and quality of dietary fats, carbohydrates, and proteins), alcohol and other bioactive dietary components (i.e., polyphenols), as well as the effect of different dietary patterns. The possible mechanisms behind the metabolic effects of each dietary component were also discussed.

## Introduction

For many years, abnormalities in postprandial lipemia (PPL), particularly those related to triglyceride-rich lipoproteins (TRL), have been considered an independent cardiovascular risk factor, even more important than altered fasting triglyceride (TAG) metabolism (1).

PPL may be influenced by different factors, but among these, diet and its respective components represent the main modulators. However, there are few human intervention studies focusing on this topic, and these have yielded very different results. This heterogeneity depends on several factors, including type of study population (healthy individuals, obese people, individuals with different metabolic diseases), genetic background, type and composition of test meals used to evaluate PPL response, methods used to study PPL (from simple measurements of plasma TAG to more detailed evaluation of exogenous and endogenous lipoproteins), and type of nutritional intervention (acute vs. chronic, changes in energy intake and/or nutrients, use of supplements instead of natural foods). This multitude of factors makes it difficult to draw direct comparisons between the studies, and it is still more difficult to translate this scientific data into recommendations and practical implementations.

Therefore, in this review, we tried to clarify some of the more intricate aspects of this topic, focusing on chronic (short/medium and long-term) intervention studies carried out in humans, to evaluate the real impact of dietary habits on PPL. Acute studies were considered when chronic studies were not available, or to highlight possible mechanisms of action.

After a chapter providing insight into the possible mechanisms, available evidence on the impact of weight loss, as well as different nutrients (quantity and quality of dietary fats, carbohydrates and protein), alcohol, and other bioactive dietary components (i.e., polyphenols) on PPL was reviewed, excluding intervention studies with supplements. Moreover, we looked at the effect of different dietary patterns and tried to consider, wherever possible, the confounding factors reported above.

## Postprandial Lipemia: Pathophysiological Premises for Dietary Impact

Postprandial lipemia consists of changes in plasma lipid concentrations and composition occurring after a meal. The main constituents of these changes are the triglyceride-rich lipoproteins (TRLs). TRLs have both intestinal (chylomicrons) and hepatic (very low-density lipoprotein, VLDL) origin. Chylomicrons are characterized by the presence of one molecule of apolipoprotein B48 (ApoB48), while the lipoproteins of hepatic origin instead contain one molecule of apolipoprotein B100 (ApoB100). Postprandial variations in lipoprotein concentration/composition depend on 1) uptake of dietary fat by the gut and secretion rate of chylomicrons, 2) intra-vascular clearance of TRLs and hepatic re-uptake of remnants, and 3) hepatic lipid metabolism and secretion rate of VLDL. Balance between these different pathways is finely regulated by insulin. In conditions related to insulin resistance (e.g., metabolic syndrome, obesity, type 2 diabetes), lipolysis in adipose tissue is increased, making more substrate available for the synthesis of hepatic triglycerides. This, along with reduced inhibition of liver catabolic pathways, induces a more pronounced secretion of VLDL. At the intestinal level, insulin resistance favors intracellular apo B48 stability and promotes activity in microsomal triglyceride transfer protein, leading to an increase in chylomicron production (2, 3). Thus, in the postprandial phase, intravascular clearance, which is mediated by lipoprotein lipases, is overloaded by contemporary increases in intestinal and hepatic TRLs. This competition increases TRL remnant production that, in turn, competes for receptor-mediated re-uptake by the liver. In conditions of insulin-resistance, the consequent inefficient clearance results in a prolonged postprandial lipemic response (4).

Quantitative and qualitative changes in diet may interfere with each step of postprandial lipid metabolism in both physiological and non-physiological conditions (Table 1).

**Table 1:** Possible dietary impact on metabolic pathways regulating postprandial lipemia.

**Dietary components**	**Metabolic pathways**		
	**Chylomicrons production**	**TRLs clearance**	**VLDL production**
Caloric restriction and weight loss	↓	↑High LPL activity	↓ Less substrate for TG synthesis
Total fat	↑	↑	↑
MUFA	↑ Early ↓ Late	↑High LPL activity and/or Low Apo CIII	↓
PUFA	↓	–	↓ Highly oxidized in the liver
SFA	–	–	↑
Fructose	↑	↓High Apo CIII	↑
Fiber	↓	↑	↓
Proteins	↓	–	–
Polyphenols	↓	–	↓
Alcohol	–	↓	↑

*↓, reduced activity; ↑, enhanced activity; –, no relevant effects; TRLs, triglyceride-rich lipoproteins; TG, triglycerides; MUFA, monounsaturated fatty acids; LPL, lipoprotein lipase; PUFA, polyunsaturated fatty acids; SFA, saturated fatty acids; Apo, apolipoprotein*.

As for the first step, caloric restriction directly reduces chylomicron production by reducing the availability of nutrients to be absorbed and, indirectly, by improving insulin resistance. At the intestinal level, improved insulin sensitivity decreases chylomicron production (2).

Among the different types of nutrients, carbohydrates and fats are the principal inducers of postprandial lipemia.

In relation to carbohydrates, according to mechanistic *in vivo* and *in vitro* studies by Lewis and colleagues (5), monosaccharides indirectly induce an increase in postprandial plasma lipid levels by stimulating intestinal *de novo* lipogenesis (in particular fructose) and chylomicron secretion, through the rapid mobilization of intracellular lipid droplets and preformed chylomicrons. Conversely, at the intestinal level, dietary fibers may exert a triglyceride-lowering effect by disrupting the micellization process, altering gut motility, physically impeding fat absorption through the formation of a water barrier between nutrients and intestinal mucosa, and by altering gut microbiota (6). Other evidence also suggests that dietary fibers may alter chylomicron secretion (6, 7) (Table 1).

As for fats, acute studies in humans have shown a direct relationship between the amount of fat ingested and the corresponding increase in circulating chylomicrons (Table 1). However, the size and shape of this increase may depend on the type of fat and the way in which it induces chylomicron production. Indeed, according to kinetic studies (8), monounsaturated fatty acids (MUFAs) are preferentially and more rapidly absorbed at the intestinal level compared to other types of fats.

In addition, proteins may interfere with the secretion of intestinal TRLs by slowing down the gastric emptying rate and consequently reducing fat absorption in the intestine (9). Similarly, polyphenols have been shown to inhibit pancreatic lipase, thus reducing triglyceride (TAG) digestion and absorption in the intestine (10) (Table 1).

Dietary components may also regulate intravascular clearance of TRLs by modulating enzymatic activities involved in this process, such as lipoprotein lipase and/or its inhibiting or activating cofactors, Apo CIII or Apo CII. Qualitative and quantitative dietary changes may also influence this pathway by regulating insulin sensitivity. Indeed, lipoprotein lipase activity is reduced in insulin-resistant conditions, while it is enhanced by insulin. For this reason, caloric restriction, and consequent body weight reduction and adipose tissue redistribution, may reduce postprandial lipemia by favoring lipoprotein lipase activity and reducing Apo CIII concentrations through the improvement of insulin sensitivity (11) (Table 1).

As for the effects of different macronutrients, carbohydrates do not seem to dramatically influence intravascular lipoprotein clearance. Some reports show no influence on lipoprotein lipase activity after a high-carbohydrate diet (12) and no changes in Apo CIII plasma or lipoprotein concentrations (13). However, recent data show that the triglyceride-raising effects of fructose mainly depend on the increased postprandial concentrations of Apo CIII in plasma and TRLs (14). This would be related to the lack of stimulation of insulin secretion by fructose, and the consequent lack of inhibition of Apo CIII expression. Among carbohydrates, dietary fibers may enhance the clearance of TRLs through the improvement of insulin sensitivity (6) (Table 1). Dietary fats are able to interact more effectively with intravascular clearance systems (Table 1). Diets rich in MUFAs have been shown to produce chylomicrons more prone to lipolysis (15) and stimulate the activity of lipoprotein lipases (16), likely through an improvement in insulin sensitivity (17). This enhanced clearance of triglyceride-rich lipoproteins produced after a MUFA diet has also been shown in the study of Zheng C et al. (18) in which the MUFA diet resulted in a ≈4–6-fold increase in secretion of VLDLs and IDLs containing both apo E and apo C-III. These lipoproteins are mostly cleared from the circulation and are minor precursors of LDL.

Among other nutritional factors, alcohol has also been shown to inhibit lipoprotein lipase and affect clearance of TRLs (19).

The stimulation of hepatic lipogenesis and/or the inhibition of lipid oxidation, and consequent increased synthesis and secretion of lipoproteins of hepatic origin, seems to be the main pathway leading to an increase in plasma triglyceride levels after high-carbohydrate diets, especially those rich in starch and simple sugars. This has been highlighted by kinetic studies evaluating the relative contribution of VLDL-triglyceride assembly, production, and clearance. Although not univocally (20), these studies show that short-term interventions with high carbohydrate diets induce an increased production of VLDL and a reduction in fatty acid oxidation (21–23). This suggests that increased postprandial triglyceride levels observed after this type of diet are mediated by the increase in hepatic production of triglyceride-rich lipoproteins driven by the excess of substrate availability. The increase in hepatic *de novo* lipogenesis has also been suggested by some authors to be the main pathway mediating postprandial hypertriglyceridemia in fructose overfeeding, especially in people with non-alcoholic fatty liver disease (24). Similar effects have been observed with alcohol, which promotes hepatic triglyceride synthesis and VLDL production by stimulating adipose tissue lipolysis and providing a substrate for *de novo* lipogenesis in the form of a product of hepatic alcohol oxidation (α-glycerolphosphate). Changes in cholesteryl ester transfer protein (CETP) activity have been suggested as another possible pathway through which alcohol may influence postprandial lipid response, particularly in terms of lipoprotein composition. However, the few studies reporting data on postprandial lipemia do not substantiate this hypothesis (25).

Additionally, quality of fats may influence the production of VLDL by differentially regulating their partitioning among catabolic or lipogenic hepatic pathways. In particular, MUFAs and PUFAs are preferentially oxidized compared to SFAs (26) (Table 1).

## Effects of Weight Loss on Postprandial Lipemia

Weight loss in overweight/obese individuals is certainly the main strategy that can have a positive effect on fasting TAG levels (27). Less is known about the influence of weight loss on PPL, either because the studies available are scant, or due to the difficulty in delineating the magnitude of beneficial effects of body weight loss on PPL from those related to fasting lipemia.

Nonetheless a certain variability, evidence derived from clinical trials in humans suggests that consistent body weight loss (10% or more) after a medium-term intervention, from 6 to 16 weeks, reduces PPL in overweight/obese patients (Table 2).

**Table 2:** Short/medium-term controlled studies on the effects of weight loss on postprandial lipemia.

**Reference**	**Study population**	**Study design**	**Intervention (% TEI)**	**Duration**	**Challenge**	**Outcomes PPL**
(28)	40 Obese M/W with atherogenic dyslipidemia	Randomized parallel group	Energy-restricted low-CHO diet (12% CHO, 59% F, 28% P) vs. energy-restricted low-Fat diet (56% CHO, 24% F, 20% P)	12 weeks	Oral fat tolerance test	↓ TAG tAUC adjusted for fasting values after the low-CHO diet,
(29)	22 Obese M MetS with atherogenic dyslipidemia	Randomized parallel group	Energy-restricted diet vs. maintenance weight diet	16 weeks	Oral fat tolerance test	= apo B48 tAUC and iAUC ↓ retinil palmitate tAUC after the energy-restricted diet
(30)	15 Overweight M	Randomized cross-over	Energy-restricted very low-CHO diet (8% CHO, 62% F, 28% P) vs. energy-restricted very low-Fat diet (56% CHO, 24% F, 20% P)	6 weeks	Oral fat tolerance test	↓TAG tAUC greater after the very low-CHO diet
(31)	13 Overweight W	Randomized cross-over	Energy-restricted very low-CHO diet (9% CHO, 63% F, 28% P) vs. energy-restricted very low-Fat diet (59% CHO, 21% F, 20% P)	4 weeks	Oral fat tolerance test	↓TAG tAUC similar after both diets
(32)	13 Overweight/Obese M/W	Randomized cross-over	Intermittent energy-restricted diet (38% CHO, 36% F, 26% P) vs. continuous energy-restricted diet (38% CHO, 36% F, 26% P)	10 weeks	Oral fat tolerance test	↓TAG iAUC after the intermittent energy-restricted diet

*TEI, total energy intake; PPL, postprandial lipemia; ↓, reduction; =, no changes; M, men; W, women; CHO, carbohydrates; F, fats; P, proteins; TAG, triglycerides; tAUC, total area under the curve; MetS, metabolic syndrome; iAUC, incremental area under the curve*.

In fact, in forty overweight/obese individuals with moderately elevated fasting TAG (1.69–5.65 mmol/l) following a 12-week low-calorie low-carbohydrate [12% of total energy intake (TEI)] or low-fat diet (24% of TEI), the postprandial total area under the curve (tAUC) TAG reduction was significantly greater after the very low-carbohydrate diet (−47%) than after the low-fat diet (−15%), and the tAUC normalized for fasting TAG remained significantly lower. Importantly, despite similar calorie reductions, weight loss in the low-carbohydrate group was greater than that in the low-fat control group (−10.5 vs. −5.5%), and this difference may explain the greater reduction in TAG response observed in the low-calorie-low-carbohydrate diet (28). Aside from having a short duration and small sample size, the high degree of dietary compliance strengthened the study.

Moreover, a 16% reduction in TEI for 16 weeks in abdominally obese men with moderately elevated TAG, leading to a weight loss of 10 kg on average, reduced fasting (−17.5%) TAG and improved insulin sensitivity, without changes in incremental area under the curve (iAUC) and tAUC for ApoB48, but with a significant decrease in retinyl palmitate tAUC, suggesting that weight loss might not affect the number of chylomicrons but instead their lipid composition (29).

In addition to the different extents of weight reduction, is the type of hypocaloric diet important in influencing PPL?

This point is strictly related to the use of a very low-carbohydrate diet, namely a ketogenic diet (Table 2). In this regard, a ketogenic diet (carbohydrate <8–10% of TEI) followed for 6 weeks was able to reduce fasting TAG (-44%) in overweight men compared to a low-fat diet. Instead, the postprandial TAG tAUC was reduced after both diets, but the decrease was significantly higher after the ketogenic diet (−38 vs. −19%). This effect was associated with a statistically significant but modest body weight reduction, achieved only at the end of the ketogenic diet, which may justify the greater reduction in postprandial TAG (30). As reported previously, a similar ketogenic diet seems to induce more remarkable effects on postprandial response in hypertriglyceridemic individuals if associated with greater weight loss (28). However, not all the studies are concordant. In fact, a ketogenic diet followed for 4 weeks resulted in a similar decrease in postprandial TAG (−29%) compared to a low-fat diet (−25%) in overweight women (31). In this case, due to the very short duration of the study, the weight reduction was insignificant following both diets.

Another relevant question is related to the possibility that the energy restriction model may have different effects on the PPL beyond body weight reduction and the magnitude of energy restriction. Only one study reporting data on PPL has dealt with this issue: an intermittent energy restriction (based on 2 consecutive days of very-low energy formula-based meals and 5 days of euenergetic healthy diet) reduced by 40% incremental postprandial TAG, without changes in fasting TAG compared with a continuous low-calorie model with the same level of body weight reduction (−5%), achieved on days 59 and 73 in the two groups (32) (Table 2). An important limitation of the study was the small sample size and the higher attrition rate with intermittent energy restriction.

From the available data, it seems that weight loss may improve PPL in overweight/obese individuals with and without lipid abnormalities. This effect seems to be, at least in part, independent of the effect on fasting TAG, and in relation to the magnitude of weight loss. Moreover, a low-calorie regimen very low in carbohydrate intake seems more effective, although not all the studies are concordant; furthermore, it is important to underline that the duration of trials performed was short (4–16 weeks), and whether these effects are lost with time—as generally happens for fasting TAG in long-term studies (24 months of follow-up)—is still unknown (27).

In relation to the possible mechanisms, the few intervention studies carried out in humans seem to confirm the role of improved insulin resistance with a consequent positive impact on TRL clearance and VLDL production, as discussed previously (2, 11, 33) (Table 1).

## Effects of Carbohydrates and Fats on Postprandial Lipemia

Carbohydrates and fats account for the main proportions of macronutrient intake across different cultural backgrounds and are the principal inducers of PPL. Postprandial increase in plasma TAG is linearly related to the amount of ingested fats, as shown in acute studies in which proteins and carbohydrates were kept constant, and fats were gradually added to different test-meals. This relationship, independent of the type of fat used, was consistent with a wide range of fat intake with a lower threshold at 15 g of fat (34–36).

Carbohydrates, when added to a fat load, also increase postprandial TAG. However, this effect differs according to the type of carbohydrate added. For simple sugars, while the addition of glucose (50–100 g) inconsistently determined an increase in postprandial TAG in different studies (34, 37), 75 g of fructose (38, 39) or sucrose (40) resulted in a dramatic increase in PPL in healthy people. However, more studies are needed to conclude that fructose or sucrose has more adverse effects than glucose. Conversely, carbohydrates from starchy food did not influence PPL in healthy people (41). In addition, postprandial TAG increased linearly with the glycemic index of different foods containing the same amount of carbohydrates in individuals with insulin resistance (42). It is noteworthy that adding carbohydrates (40% starch, 46% sucrose, and 14% lactose) to a fatty meal delays the plasma TAG peak (7, 43).

Hence, both fats and carbohydrates were able to elicit a PPL response in experimental settings where these nutrients were added, increasing the energy content of the meals. This differs from real-life conditions where carbohydrates and fats substitute each other in different relative amounts; therefore, both their respective proportions may influence PPL. A recent meta-analysis (44) evaluated the effects of an isocaloric exchange between carbohydrates and fats by pooling acute studies in which carbohydrate-rich meals were compared with high-fat meals that differed by <10% in total energy intake (TEI) and <20% protein content. In this meta-analysis, each 10% increase in energy in fat-replacing carbohydrates determined an increase in mean postprandial TAG of 0.06 mmol/l. This finding would suggest, at least in acute conditions, a more powerful lipemic effect for fats than for carbohydrates.

No similar analysis is available for non-acute studies, although several papers report the effects of short/medium-term trials comparing low-fat/high-carbohydrate (LF-HCHO) diets with high-fat/low-carbohydrate diets (HF-LCHO) (Table 3). In the experimental models in which the tested diets largely differed for the relative amounts of carbohydrates and fats (from 10 to 60% TEI), LF-HCHO diets induced a higher and prolonged postprandial TAG response to a fat load compared with a HF-LCHO diet (45, 52). Notably, postprandial TAG response was strictly related to the “chronic” dietary effects on fasting plasma TAG, which were higher after the high-carbohydrate diets. In fact, the differences in postprandial plasma TAG were observed when calculated as tAUC but not when calculated as iAUC (45, 53). This effect of dietary carbohydrates has also been observed with dietary interventions, with fewer differences in the relative amounts of carbohydrates and fats (55 and 30% in LF-HCHO and 45 and 40% TEI in HF-LCHO diet). In this study (12), plasma concentrations of retinyl palmitate were not different between the two diets over the postprandial phase, suggesting that the increase in plasma TAG with LF-HCHO diet was due to an increased production of VLDL rather than of lipoproteins of intestinal origin. Other studies (46, 47) comparing dietary interventions moderately differing in the amounts of carbohydrates and fats failed to find any difference in postprandial TAG or lipoprotein composition between diets.

**Table 3:** Isoenergetic short/medium-term studies on the effects of dietary carbohydrate quality and quantity, and of dietary fat amount on postprandial lipemia.

**Reference**	**Study population**	**Study design**	**Intervention**					**Duration**	**Challenge**	**Outcomes postprandial TAG increment**
				**CHO *%TEI***	**Sugars *%TEI***	**Fiber *g/day***	**Fats *%TEI***			
(45)	10 Healthy W	Randomized cross-over	Low-CHO	10	3	12	60	4 weeks	Oral fat tolerance test	High-CHO equal to low-CHO
			High-CHO	55	25	20	25			
(12)	10 T2D M	Randomized cross-over	High-MUFA	40	17	26	45	6 weeks	Oral fat tolerance test	High-CHO equal to low-CHO
			High-CHO	55	23	36	30			
(46)	88 M/W dyslipidaemia and/or IR	Cross-over	Low-MUFA	49	18	22	37	7 weeks	High-fat test meal	Low-MUFA equal to high-MUFA equal to high-CHO
			High-MUFA	49	18	22	36			
			High-CHO	54	19	34	30			
(47)	12 T2D	Randomized cross-over	High-MUFA	45	n.r.	25	40	6 weeks	High-fat test meal	High-MUFA equal to high-CHO
			High-CHO	55	n.r.	25	30			
(48)	8 post-obese 10 healthy W	Randomized cross-over	Low-CHO	41	2	32	46	2 weeks	Breakfast, lunch and dinner with the same composition of the followed diet	Low-CHO greater than high-sugar and high-starch
			High-starch	59	2	22	28			
			High- sugar	59	23	20	18			
(49)	18 T2D	Randomized cross-over	High-MUFA	45	10	16	37	4 weeks	Test-meal with the same composition of the followed diet	High-MUFA greater than high-fiber
			High-fiber	52	10	54	30			
(50)	45 T2D	Randomized parallel groups	High-MUFA	40	10	16	42	8 weeks	Test-meal with the same composition of the followed diet	High-MUFA greater than high-fiber
			High-fiber	53	10	52	28			
(51)	65 M/W MetS	Randomized parallel groups	Whole-grain	51	n.r.	20	32	12 weeks	Test-meal with the same composition of the followed diet	Refined-grain greater than whole-grain
			Refined-grain	50	n.r.	40	33			

*CHO, carbohydrates; TEI, total energy intake; TAG, triglyceride; W, women; T2D, type 2 diabetes; M, men; IR, Insulin Resistance; n.r., not reported; MetS, Metabolic Syndrome*.

Of relevance, in all studies showing more powerful lipemic effects for LF-HCHO, simple sugars represented a relevant proportion (20–30 % of TEI) of carbohydrates (45, 52, 53).

Interestingly, in 3-day, 70% carbohydrate dietary interventions differing in the relative amount of simple sugars (sucrose) and starchy foods, Culling et al. (53) showed that a sucrose-rich diet induced a more pronounced increase in fasting and postprandial plasma TAG than a starchy-diet. These effects after a very short dietary intervention have also been observed after chronic adaptation. In fact, another study with a longer follow-up (48) showed a higher total postprandial TAG AUC after high-fat and high-sucrose diets than after a high-starch diet, and a higher TAG iAUC after the high-fat diet than after the higher starch and sucrose diets. This suggests that carbohydrate quality is a main determinant of PPL, independent of carbohydrate amount.

Carbohydrate quality may depend on different factors such as the type of carbohydrate (simple vs. starch, types of simple sugars) and the amount and type of dietary fibers.

### Simple Sugars

Evaluating the impact of the type of simple sugars on PPL mainly entails, in particular, a comparison between glucose and fructose.

Wang et al. (54) specifically meta-analyzed the effect of an isocaloric and hypercaloric substitution of glucose for fructose in short-term studies, finding no effects in the isocaloric trials and a significant postprandial TAG-rising effect in studies of hypercaloric diets. In the study by Matikainen et al. (55), which was not included in this meta-analysis, where 12 weeks of daily 75 g fructose were added to an *ad libitum* food intake, a mixed-meal test resulted in an earlier rise in postprandial TAG, with a significantly greater postprandial TAG tAUC. The increase in postprandial TAG corresponded to higher ApoB48 concentrations and increased liver fat content (56).

### Dietary Fibers

Data on the TAG-lowering effect of dietary fiber come first from acute studies or short-term trials testing fiber supplementation (57). These studies consistently show that adding fiber to meals with different compositions can blunt the postprandial TAG response. Other studies have evaluated the effects of fiber from natural carbohydrate-foods in medium-term experiments (Table 3). In these trials, a daily intake of dietary fiber ranging between 30 and 52 g reduced postprandial TAG response to a test-meal with a composition similar to the diets tested, when compared with a diet low in fiber and with a high glycemic index or a diet rich in monounsaturated fat (MUFA) (49, 50, 58). The TAG-lowering effect of dietary fibers from natural food was evident with diets either extremely (≈63 % TEI) (58) or moderately (≈50 % TEI) rich in carbohydrates (49, 50). Interestingly, a high-carbohydrate/high-fiber diet did not increase fasting TAG and induced a smaller incremental postprandial TAG response compared to a MUFA diet (49, 50) in patients with type 2 diabetes. The lower postprandial TAG response was mainly related to a lower TAG in chylomicrons and a large VLDL. Foods utilized in these diets were rich mainly in soluble fiber, generally considered more active in glucose and lipid metabolism. However, according to recent evidence, insoluble fiber is able to act on PPL, as shown by the relevant reduction of the average postprandial TAG response (−43%) after a whole-grain diet, particularly rich in insoluble fiber, compared to a refined grain diet with a lower amount of insoluble dietary fibers (51) (Table 3).

These data suggest that the metabolic effects of dietary fibers may be linked not only to the type (soluble vs. insoluble) but also, and perhaps more importantly, to other characteristics such as their fermentability (59).

All the results reported above suggest that the effect of carbohydrates on PPL is strictly related to the type and quality of carbohydrates. In fact, different foods with the same amount of carbohydrates could have different nutritional and structural properties that impact on PPL more than carbohydrates *per se*.

Overall, the evidence described above would suggest that a diet with a moderate amount of carbohydrates from starchy foods rich in fiber would be preferable to a diet moderately high in fats for preventing an increase in postprandial TAG.

### Quality of Fat

Several studies (15–17, 60) have compared the short/medium-term effects of dietary interventions with a balanced proportion of carbohydrates (40–45% TEI) and total fats (30–35% TEI) differing only in the proportion of saturated fats (SFAs) (17–22% TEI) and MUFAs (18–24% TEI) (Table 4). These trials consistently showed no differences in the overall postprandial TAG response, but an increased early response with a consequent late decrease following diets rich in MUFA compared with diets rich in SFA (60).

**Table 4:** Isoenergetic short/medium-term studies on the effects of fat quality on postprandial lipemia.

**Reference**	**Study population**	**Study design**	**Interventions**						**Duration**	**Challenge**	**Outcomes postprandial TAG and lipoprotein increment**
				**CHO *%TEI***	**Fats *%TEI***	**SFA *%TEI***	**MUFA *%TEI***	**PUFA *%TEI***			
(16)	12 T2D	Randomized cross-over	High-SFA	46	37	17	15		3 weeks	Standard High-Fat Test-meal	Early chylomicron TAG: high -MUFA greater than high -SFA
			High-MUFA	46	37	8	23				
(17)	162 healthy	Randomized parallel groups	High-SFA	44	37	18	13	4.7	12 weeks	Standard high-fat test-meal	Plasma TAG^*^: high-MUFA equal to high -SFA
			High-MUFA	46	37	10	21	4.6			
(15)	23 healthy	Randomized cross-over	High-SFA	43	41	17	14	7.0	8 weeks	Standard high-fat test-meal	Early plasma TAG: high -MUFA greater than high -SFA
			High-MUFA	44	41	12	18	7.2			
(60)	20 M	Randomized cross-over	High-SFA	47	38	22	24	4	4 weeks	High-fat meal with the same composition of the followed diet	TRL particles number: high -MUFA lower than high -SFA TRL particles size: high -MUFA greater than high -SFA
			High -MUFA	47	38	<10	24	4			
			High -PUFA	55	<30	<10	12	8			
(61)	32 M	Randomized parallel groups	High -SFA	49	37	17	12	4	29 days	High-fat meal with the same composition of the followed diet	TAG ApoB48 iAUC: high-PUFA greater than high -SFA
			High -PUFA	45	40	10	14	13			
(62)	8 healthy	Randomized cross-over	High-SFA	43	42	28	12	2.1	25 days	Vitamin A fat-loading test	RP chylomicron high -PUFA lower than high -SFA RP non-chylomicron high -PUFA lower than high -SFA
			High-PUFA n-6	43	42	13	13	18			

*CHO, carbohydrates; TEI, total energy intake; SFA, saturated fatty acid; MUFA, monounsaturated fatty acid; PUFA, polyunsaturated fatty acid; PPL, postprandial lipemia; T2D, type 2 diabetes; n. a., not available; TAG, triglyceride; M, men; TRL, Triglyceride Rich Lipoproteins; iAUC, incremental area under the curve; RP, Retinil Palmitate*.

* *no differences in fasting TAG levels*.

In all studies, this early increase in plasma TAG was consistent with an increased TAG in lipoproteins of intestinal origin. However, not all studies consistently showed an increase in the number of chylomicrons (15), with some trials reporting an increase and others a reduction in ApoB48 concentration (16), linearly related to the amount of MUFAs ingested (63). In support of the production of larger, less atherogenic chylomicrons, instead of a change in the number of particles, there is also evidence of a larger TRL after a 4-week MUFA diet compared to a SFA-diet measured by magnetic resonance spectroscopy (60).

Very few studies comparing the TAG-rising effects of diets rich in SFA or n-6 and/or n-3-PUFA from natural foods have led to conflicting results. Two trials have yielded particularly relevant insights, since they were designed to specifically distinguish acute and chronic postprandial effects from these types of fats. Bergeron et al. (61) found that postprandial plasma TAG, ApoB48, and ApoB100 were higher after an SFA-diet than after an n-6 PUFA diet. However, PUFA-rich meals within the context of high SFA- and high n-6-PUFA-diets determined a greater percent increase at 3 h in TAG, ApoB48, and ApoB100, compared to the SFA-meal. Conversely, an SFA meal resulted in a prolonged increase in ApoB100 concentrations that fell below the post-absorptive values 9 h after meal. These findings suggest that a PUFA-diet would acutely stimulate the production of lipoproteins of intestinal origin, while the SFA-diet would chronically stimulate the production of lipoproteins of hepatic origin, determining a prolonged postprandial increase in plasma TAG. In contrast with these results, Weintraub et al. (62) observed, compared to a high SFA-diet, a significantly greater reduction of plasma TAG after a PUFA n-3 supplementation (3.5 g/1,000 Kcal) and a PUFA n-6 rich-diet. This reduction was paralleled by a reduction of 41% after a PUFA n-6 diet and 68% after PUFA n-3 supplementation of retinyl-palmitate concentrations in chylomicron and non-chylomicron particles, respectively.

As the effects of supplements fall outside the aims of this review, we only mention, for the sake of completeness, that extensive literature is also available regarding the effects of supplementation with n-3 PUFA. Overall, evidence is in favor of a reduction of PPL by n-3 supplementation, partly linked to the effects on fasting TAG (17). Nonetheless, there is no complete consistency in the findings among trials with different study designs and populations (57).

## Effects of Other Dietary Components on Postprandial Lipemia

Among other dietary components that might affect PPL, it is worth mentioning proteins, polyphenols, and alcohol.

Protein quantity and quality have shown a pivotal role in the reduction of TAG response, at least in acute settings and with protein supplements. Overall, no significant differences in fasting TAG were detected in all studies investigating protein quality or quantity, suggesting that the possible effect is related to the postprandial period. As for PPL, the addition of casein (45–50 g) during an 8-h high fat meal challenge (80 g of fat) significantly reduced the TAG response (evaluated as 8 h-iAUC) in healthy volunteers and in individuals with type 2 diabetes (41, 64). However, no difference was detected for TAG in the VLDL-fraction or whole plasma (41, 64). These results have been confirmed in the medium-term, although only one study is available to date (Table 5). Indeed, a 6-week nutritional trial has demonstrated that substituting carbohydrates with protein (mainly from lean red beef, veal, and lamb) in the habitual diet can influence chylomicron metabolism in moderately hypertriglyceridemic subjects. In particular, a high protein diet (25% protein, 35% carbohydrate, 30% fat, and 10% alcohol) significantly reduced ApoB48 response to an oral fat meal challenge (evaluated as 8 h-iAUC) compared to a low protein diet (14% protein, 53% carbohydrate, 30% fat, and 3% alcohol) (65). However, due to the differences in other nutrients, such as carbohydrates, it is difficult to ascribe the effect on PPL to only the protein content.

**Table 5:** Short/medium-term controlled studies on the effects of protein, polyphenol, alcohol, and dietary patterns on postprandial lipemia.

**Reference**	**Study population**	**Study design**	**Intervention (% TEI)**	**Duration**	**Challenge**	**Outcomes PPL**
**Protein**						
(65)	20 M/W with moderate hyperTAG	Randomized parallel group	Isocaloric low protein diet (14%P, 53%C30% F) vs. isocaloric high protein diet (25%P, 35%C, 30%F)	6 weeks	Oral fat tolerance test	= iAUC TAG↓ iAUC ApoB48 after high protein diet
(66)	52 overweight M/W	Randomized 2 × 2 factorial design	Isocaloric diet vs. isocaloric diet + 60 g whey protein	12 weeks	High Fat test meal	= plasma TAG response ↓ iAUC ApoB48 after whey protein diet
**Polyphenols**						
(67)	78 overweight M/W MetS	Randomized parallel group	Low-polyphenol isocaloric diet (~350 mg/day) vs. high-polyphenol isocaloric diet (~3,000 mg/day)	8 weeks	High Fat test meal	↓ tAUC plasma TAG ↓ tAUC large VLDL TAG = iAUC plasma TAG = iAUC large VLDL TAG
**Alcohol**						
(25)	12 healthy W	Randomized cross-over	Red wine (30 g/day of alcohol) vs. red grape	3 weeks	High fat dinner	↑TAG tAUC in the red wine group
(81)	12 healthy M	Randomized cross-over	red wine or beer or spirits (40 g/day of alcohol) vs. mineral water	3 weeks	High fat dinner	↑TAG tAUC after the red wine, beer and spirits
**Dietary Patterns**						
(60)	20 healthy M	Randomized cross-over	Western diet (47% CHO, 15% P, 38% F) vs. Mediterranean diet (47% CHO, 15% P, 38% F) vs. high CHO enriched with ALA diet (55% CHO, 15% P, <30% F)	4 weeks	High fat breakfast	↓ TRL tAUC after the Mediterranean diet* ↑ TRL size after the Mediterranean diet
(68)	135 overweight/ Obese M/W	Randomized parallel group	Mediterranean diet (50% CHO, 12–15% P, 35–38% F) vs. AHA low-Fat diet (55–60% CHO, 15% P, <30% F)	3 months	Oral fat tolerance test	↓ ApoB48 iAUC greater after the Mediterranean diet
(69)	241 without T2D 316 T2D M/W	Randomized parallel group	Mediterranean diet (50% CHO, 15% P, 35% F) vs. low-Fat diet (55% CHO, 15% P, <30% F)	3 years	Oral fat tolerance test	↓ cholesterol in remnant TRL after the Mediterranean diet in T2D

TEI, total energy intake; PPL, postprandial lipemia; M, men; W, women; CHO, carbohydrates; F, fats; P, proteins; =, no changes; iAUC, incremental area under the curve; ↓, reduction; ↑, increase; TAG, triglycerides; tAUC, total area under the curve; MetS, metabolic syndrome; VLDL, very low-density lipoprotein; AHA, American Heart Association; ALA, α-linolenic acid; TRL, triglycerides rich lipoproteins; T2D, type 2 diabetes. *no differences in fasting TAG levels.

As for protein quality, most of the studies have focused on the effect of whey protein against casein on PPL within an isocaloric meal (70–73). Interestingly, the addition of whey protein had a hypotriglyceridemic effect in the postprandial period, particularly in the chylomicron fraction, when compared to casein.

As for the addition of other protein sources (72–74), that is soy, cod, or gluten protein, only soy protein induced a greater reduction of TAG compared to casein during an 8-h-high fat-meal challenge (74). The effect of milk-derived protein on PPL has also been tested in the long-term, specifically in a 12-week isocaloric nutritional trial (66) with a 2 × 2 factorial design aiming to highlight whether milk protein (whey or casein) and fats (high or low medium-chain fatty acids) can act differently as drivers of abnormalities in TAG response in overweight/obese individuals (Table 5). For protein, participants were asked to consume a protein supplement (60 g) as whey or casein powder ready to mix with water during the day. After 12 weeks, whey protein supplementation markedly reduced the Apo B48 response during a 6 oral fat challenge (evaluated as iAUC) as compared to casein, and independently from the amount of medium-chain fatty acids.

In recent years, polyphenols have received much attention due to their association with lower cardiovascular risk in observational studies. Among cardiovascular risk factors, the effect of polyphenols on PPL has been poorly explored, at least in medium- and long-term human studies (75). To the best of our knowledge, there is only one medium-term clinical trial (67) evaluating the effect of a polyphenol-rich diet (~3 g/day) on PPL in overweight/obese individuals at high cardiometabolic risk (Table 5). After 8 weeks, a significant reduction in both fasting and postprandial TAG response, mainly in the large VLDL fraction, was observed after a 6-h oral fat challenge. More specifically, plasma and large VLDL-TAG were significantly affected by polyphenol at fasting and in the postprandial period (evaluated as tAUC), whereas no effect was detected on the increment in the postprandial phase (iAUC). In addition, no changes in chylomicron-TAG and Apo B48 concentrations were observed (76). Among the different phenolic subclasses, the hypotriglyceridemic effect was mainly related to flavanone intake (62 g/day) (77). However, further chronic studies are needed to demonstrate the postprandial hypotriglyceridemic effect of polyphenols in the medium and long term, and whether this effect is independent of fasting TAG metabolism.

Conversely, there is ample evidence that alcohol has detrimental effects on fasting TAG, especially if consumed in moderate to high doses and in individuals with high cardiovascular risk (78).

The effects of alcohol consumption on postprandial TAG response have been evaluated in different acute (hours or 1 day) settings (79, 80), and the increase in postprandial TAG was generally lower with dealcoholized red wine compared to red wine or when red wine was compared to other alcoholic drinks.

Looking at the short/medium term effect of alcohol intake on PPL, van der Gaag et al. (25) investigated the effect of moderate consumption of red wine (30 g of alcohol/day) or red grape juice with the evening dinner for 3 weeks in premenopausal and postmenopausal women. The post-dinner TAG tAUC was significantly increased after wine consumption than after grape juice in both premenopausal and postmenopausal women (+ 202 and 113%, respectively), with no differences in the pre-dinner TAG levels or in lecithin cholesterol acyltransferase (LCAT) and CETP activity.

With respect to the possible differences between the various types of alcoholic drinks, the effects of beer, wine, spirits (a total amount of 40 g/day of alcohol) and mineral water on postprandial TAG have been evaluated in healthy men in a 3-week study. Postprandial TAG tAUC measured after dinner reached a peak at 5 h and decreased to fasting values in the next morning, with higher post-dinner concentrations vs. mineral water and without difference between beer, wine, and spirits. This suggests that the effect on PPL is linked to alcohol intake *per se* and is not modifiable by other components such as polyphenols (81).

In conclusion, there have been too few trials investigating the chronic effect of alcohol on PPL, limited by short durations and small sample sizes, to provide conclusive evidence.

Moreover, a significant component of the effect of alcohol on PPL is linked to its negative effects on the overall homeostasis of TAG.

## Effects of Dietary Patterns on Postprandial Lipemia

As discussed previously, different dietary components may influence PPL. However, individuals do not eat single nutrients or dietary components, but rather foods combined in different eating models based on a social-cultural background. The quantity, variety, or combination of different foods and beverage in a diet and the frequency with which they are habitually consumed, is defined as dietary patterns. Thus, from a clinical point of view, it is important to know the possible effects of dietary patterns on PPL.

Very few clinical trials have addressed this issue. Among the dietary patterns, the Mediterranean diet has attracted much interest in recent decades for the prevention and treatment of cardiometabolic risk factors, and some evidence of its beneficial effects on PPL is available (Table 5).

Perez-Martinez et al. (60) evaluated the effects of a 4-week Mediterranean diet, a Western diet, and a high-CHO diet enriched in α-linolenic acid on postprandial TRL (chylomicron and large VLDL and non-chylomicron fractions) in healthy individuals following a test meal resembling the diet to which they were assigned. No significant differences were observed between the three diets on fasting TAG and TAG in large and small TRLs. In the postprandial state, the Mediterranean diet promoted a reduction in the number of TRL compared with the other diets, and an increase in TRL particle size compared to the high carbohydrate diet enriched with α-linolenic acid.

Similarly, a 3-month Mediterranean dietary pattern in overweight/obese individuals promoted a small but significant decrease in fasting TAG, a similar reduction in postprandial TAG compared to a low-fat diet (iAUC: −13.7 vs. −12.5%, respectively,), and a more remarkable difference in ApoB48 (iAUC: −74.4 vs. −41.7%, respectively), suggesting a beneficial effect of the Mediterranean diet in reducing the number of ApoB48 particles in the postprandial state (68). Data from long-term (3-year) interventions seem to confirm the beneficial effects of the Mediterranean diet on PPL, at least in patients with type 2 diabetes. In fact, Gomez-Marin et al. (69) investigated the effect of a Mediterranean diet or a low-fat diet on PPL in a group of patients (241 with and 316 without type 2 diabetes) from the CORDIOPREV study who underwent an oral fat load test. After 3 years of dietary intervention, type 2 diabetic patients showed an improvement in postprandial TAG (−17.3% in tAUC) compared to patients in the low-fat diet. Furthermore, the Mediterranean diet also decreased cholesterol concentrations in the remnant TRL. No differences were observed between diets in patients without diabetes (69). Interestingly, in a subgroup of the CORDIOPREV study, a gene-diet interaction between the *APOE* polymorphism and the type of diet was found. In fact, T-allele carriers of *APOE* in the Mediterranean intervention group showed a more significant decrease in postprandial TAG and large TRL compared with CC subjects (82).

The Mediterranean diet is characterized by a combination of different components (type of fat and carbohydrates, fiber, and polyphenols) that could beneficially affect PPL through different mechanisms, as discussed previously. These different components could also act synergistically to determine their effect on PPL (Table 1).

## Conclusions

Although PPL is a well-known independent cardiovascular risk factor, human intervention studies evaluating the impact of dietary habits on PPL are rather scarce, and some of them contain notable weaknesses to be considered when drawing conclusions. In fact, some intervention studies were carried out on relatively small numbers, making the results difficult to transfer to other and broader contexts; the general (sex and age) and metabolic (healthy, overweight/ obese individuals, patients with diabetes or dyslipidemia) characteristics of participants in different studies are quite variable and, therefore, again, the results are difficult to generalize. Another point to be considered is the type of challenge used to elicit postprandial response, varying from different oral fat tolerance tests to test meals resembling real life. Additionally, there is a shortage of long-term studies. All these factors make the comparison between studies very difficult. To overcome some of the above problems, long-term controlled studies in large samples of different populations, well-characterized for their metabolic status, are needed. In addition, mechanistic studies looking in more depth at the effect on PPL are warranted, and should focus on different lipoproteins and their subfractions, as well as on the possible mechanisms, using the new technologies available.

However, from a careful analysis of the current evidence and with great caution, it is possible to conclude that (Table 6):

1. Weight loss seems to have independent beneficial effects on PPL, which is strictly linked to the magnitude of weight reduction. In the short/medium term, very low carbohydrate diets seem to be more effective, but long-term studies are needed.
2. Studies evaluating the impact of carbohydrates and fat both in terms of amount and quality are the most numerous, although the results are not always concordant and are, in some cases, very intricate. In any case, we could attempt to summarize the available evidence as follows: (a) carbohydrate quality is more important than amount in modulating PPL or, at least, its influence is in part independent of quantity; (b) in relation to carbohydrate quality/type, simple sugars, particularly fructose, increase PPL, whereas dietary fiber and fiber-rich diets decrease PPL; (c) the amount of dietary fat seems to be more important than the different types of fat in regulating PPL.
3. As for proteins, polyphenols and alcohol, the available studies are too few for any conclusions to be drawn. Moreover, most of these studies have been performed using supplements and in an acute setting. In particular, acute studies seem to indicate positive effects of polyphenols on PPL and, therefore, chronic intervention studies should be carried out to confirm these results and those derived from the only chronic intervention trial performed with a polyphenol-rich diet.
4. Among dietary patterns, the Mediterranean diet seems to induce positive effects on PPL, more by virtue of the diet as a whole than of its single components.

**Table 6:** Summary table of the dietary impact on postprandial lipemia.

	**Postprandial lipemia**
Weight-loss diet	↓
Carbohydrates:	
- Simple sugars	↑ (fructose more than glucose)
- Dietary fiber	↓↓
- Diet moderately rich in carbohydrate and fiber	↓↓
Fats:	
- Amount	↑ (acute studies)
- Quality	-/↓ MUFA vs. SFA ↓ PUFA-n6 vs. SFA
Proteins	↓ (mainly acute studies supplements)
Polyphenols	↓ (mainly acute studies)
Alcohol	↑ (mainly acute studies)
Dietary patterns	↓ (Mediterranean diet)

*↑, increase; ↓, reduction; ↓↓, strong reduction; -, no change; MUFA, monounsaturated fatty acid; PUFA, polyunsaturated fatty acids; SFA, saturated fatty acid*.

Thus, based on what we know so far, for PPL, as for the other cardiovascular risk factors and the prevention of cardiovascular disease, the dietary model to be proposed should be characterized by a high intake of fiber-rich foods, whole grain cereals, vegetables, fruit, and non-alcoholic drinks such as coffee and tea—rich in polyphenol—and fish, as well as a low intake of foodstuff rich in saturated fatty acids and simple sugars.

## Author Contributions

LB and AR conceptualized the manuscript and drafted the section regarding background. LB drafted the section regarding carbohydrate and fat. GD drafted the sections regarding weight loss and dietary patterns. CV drafted the section regarding protein and polyphenol. All authors reviewed and revised the manuscript and approved the final version.

### Conflict of Interest

The authors declare that the research was conducted in the absence of any commercial or financial relationships that could be construed as a potential conflict of interest.

## Abbreviations

Apo: apolipoprotein
CHO: carbohydrate
CETP: cholesteryl ester transfer protein
HF-LCHO: high-fat/low-carbohydrate diets
iAUC: incremental area under the curve
LF-HCHO: low-fat/high-carbohydrate diet
MUFA: monounsaturated fatty acid
SFA: saturated fatty acid
PPL: postprandial lipemia
PUFA: polyunsaturated fatty acid
TAG: triglyceride
tAUC: total area under the curve
TEI: total energy intake
TRL: triglyceride-rich lipoproteins
VLDL: very low-density lipoprotein.
